# Cross-national differences in clinically significant cannabis problems: epidemiologic evidence from 'cannabis-only' smokers in the United States, Mexico, and Colombia

**DOI:** 10.1186/1471-2458-10-152

**Published:** 2010-03-23

**Authors:** Fabian Fiestas, Mirjana Radovanovic, Silvia S Martins, Maria E Medina-Mora, Jose Posada-Villa, James C Anthony

**Affiliations:** 1 Department of Epidemiology, College of Human Medicine, Michigan State University, East Lansing, USA; 2 Department of Mental Health, Johns Hopkins Bloomberg School of Public Health, Baltimore, USA; 3 Department of Epidemiology, National Institute of Psychiatry, Mexico DF, Mexico; 4 Ministry of Social Protection and Colegio Mayor de Cundinamarca University, Bogota, Colombia

## Abstract

**Background:**

Epidemiological studies show wide variability in the occurrence of cannabis smoking and related disorders across countries. This study aims to estimate cross-national variation in cannabis users' experience of clinically significant cannabis-related problems in three countries of the Americas, with a focus on cannabis users who may have tried alcohol or tobacco, but who have not used cocaine, heroin, LSD, or other internationally regulated drugs.

**Methods:**

Data are from the World Mental Health Surveys Initiative and the National Latino and Asian American Study, with probability samples in Mexico (n = 4426), Colombia (n = 5,782) and the United States (USA; n = 8,228). The samples included 212 'cannabis only' users in Mexico, 260 in Colombia and 1,724 in the USA. Conditional GLM with GEE and 'exact' methods were used to estimate variation in the occurrence of clinically significant problems in cannabis only (CO) users across these surveyed populations.

**Results:**

The experience of cannabis-related problems was quite infrequent among CO users in these countries, with weighted frequencies ranging from 1% to 5% across survey populations, and with no appreciable cross-national variation in general. CO users in Colombia proved to be an exception. As compared to CO users in the USA, the Colombia smokers were more likely to have experienced cannabis-associated 'social problems' (odds ratio, OR = 3.0; 95% CI = 1.4, 6.3; p = 0.004) and 'legal problems' (OR = 9.7; 95% CI = 2.7, 35.2; p = 0.001).

**Conclusions:**

This study's most remarkable finding may be the similarity in occurrence of cannabis-related problems in this cross-national comparison within the Americas. Wide cross-national variations in estimated population-level cumulative incidence of cannabis use disorders may be traced to large differences in cannabis smoking prevalence, rather than qualitative differences in cannabis experiences. More research is needed to identify conditions that might make cannabis-related social and legal problems more frequent in Colombia than in the USA.

## Background

Population prevalence and global health burdens associated with drug dependence and related drug use disorders vary markedly across world regions [1]. For example, in 2003, the United Nations Office on Drugs and Crime tallied the occurrence of cannabis problems in different countries of the world, based upon administrative reporting systems from each government (UN, 2003; https://www.unodc.org/pdf/trends2003_www_E.pdf, last accessed 15 Aug 2009). According to the UN, in the Americas, the prevalence of cannabis problems for Mexico was 1%, for Peru, 2-3%, for Venezuela, 8%, and for the United States (USA), 9-10% [2]. Most likely, these wide variations in the UN estimates can be traced back to variations in administrative record-keeping of each government or to differences in study methods and instruments used [3,4]. Another possibility is that these cross-country variations are more than artifactual. In this instance, there are at least two hypotheses that might explain the observed variations.

The first of these two hypotheses is quantitative in origin: observed variations in cross-country prevalence of cannabis-related disorders might simply reflect cross-country variations in the number of people who have used this drug. The second hypothesis has to do with more qualitative between-country differences in what cannabis users experience once drug use starts. From pharmacology, we can identify some potential sources of qualitative differences in these experiences, including cannabis properties and cannabis consumption patterns, perhaps involving variations in the delta-9 tetrahydrocannabinol (THC) content of the local product [5], how it is ingested, or whether cannabis is consumed with tobacco, alcohol or other illegal drugs concurrently [6]. From the social sciences, we can point toward variations in ambient socio-demographic characteristics and socio-cultural contexts, defined to encompass laws and policies, social values, and societal dynamics. Of course, these two hypotheses are not mutually exclusive in that the sheer prevalence of cannabis smoking may drive the occurrence of cannabis dependence and other cannabis problems and, in addition, qualitative between-country differences also may shape the population-level health burdens.

The World Health Organization World Mental Health Surveys Consortium (WHO, WMHS) has created a new opportunity to investigate research questions about potential reasons for cross-site and cross-national variation in cannabis experiences. The WHMS involves a form of active surveillance, conducted by coordinated research teams that follow the same or essentially the same field survey research protocol. This approach holds constant or constrains survey research methods to be similar in order to produce cross-national and cross-site comparative data from all the participating countries, with representation of all WHO regions [7].

In this context, this study's aim is to seek evidence about actual cross-national variation in the experience of clinically significant cannabis problems (hereinafter termed "clinical features") as identified in the Spanish-American heritage populations of the USA, Mexico, and Colombia, as well as the USA general population. The focus is on general population participants who have smoked cannabis, and who may also have tried tobacco and alcoholic beverages (hereinafter, alcohol), after exclusion of cannabis smokers who had engaged in illegal use of cocaine, heroin, LSD, and other internationally regulated drugs (IRD).

The focus on 'cannabis only' users is motivated by complexities introduced when other psychoactive drug compounds are used concurrently, especially illegal drugs or those prescription drugs used extra-medically. As mentioned above, in some countries, cannabis is more likely to be consumed along with other psychoactive drugs obtained legally or illegally. To the extent that males are more likely to be engaged in such drug use patterns, an excess occurrence of cannabis problems among males might be traced back to underlying variations in use of other drugs. This possibility has prompted regression modeling with covariates and the use of matching procedures, sample restriction or exclusion rules [8]. Here, the sample restriction approach (i.e., focus on 'cannabis only' users) and individual-level matching have been applied.

The focus on the USA, Mexico, and Colombia is motivated by the fact that these were the only three WMHS Consortium countries with large Spanish-American heritage populations. To date, the only other WMH survey in the Americas has been conducted in Sao Paulo, Brasil, and the data from the Sao Paulo site are not yet available for this report's analyses.

## Methods

### Participants

The data for this research were gathered as part of the WMHS and NLAAS initiatives with community samples in Mexico (n = 5,782), in Colombia (n = 4,426), and in the USA (n = 8,228). In each country, a cross-sectional multi-stage probability sample survey design was used to designate dwelling unit respondents, who then were recruited for the survey assessment. In the USA, two complementary survey samples were recruited: (a) a sample of 5,692 community participants in the National Comorbidity Survey Replication (NCS-R); and (b) a sample of 2,554 Hispanic heritage community participants in the NLAAS.

The target population for each of these epidemiological surveys was defined to include non-institutionalized community dwelling unit residents, aged 18 years and older. In Colombia and Mexico, where about 75% of the total population resides in urban areas, the sampling frame was restricted to urban Spanish speakers, yielding a nationally representative sample for urban populations. In the USA, the NCS-R sampling frame consisted of English speaking adult household residents, yielding a nationally representative sample for both rural and urban populations (USA general population). There were no restrictions in the sampling process based in race-ethnic heritage for the NCS-R. On the other hand, the NLAAS sampling frame was restricted to adult household residents with either Hispanic or Asian-American heritage, yielding a nationally representative sample of those segments of the US population. The 2,554 Hispanic heritage NLAAS participants (hereinafter, USA Latinos) self-identified themselves as follows: 57% Mexican heritage, 10% Puerto Rican, 5% Cuban. The rest qualified as 'Other' Latino (about 28%). Participation levels exceeded 75% for each survey. Consent procedures and the protocols were approved by research ethics committees in all countries. The NLAAS and NCS-R datasets qualify as 'public use' datasets; it was necessary to secure permission from the Mexico and Colombia sites in order to bring their data into this research project on cannabis.

Among 18,454 individuals in these various nationally representative community samples, and after exclusion of 2,121 cannabis users who had been illegal users of cocaine, heroin, LSD or other internationally regulated drugs, there were 2,196 'cannabis only' users (hereinafter, 'CO' users). We did not exclude cannabis smokers who also had consumed tobacco (about 65%) or alcoholic beverages (about 99%).

### Assessment

All surveys used the WMHS version of the WHO Composite International Diagnostic Interview, version 3.0 (CIDI-WMH). The Spanish version was prepared with attention to a standard protocol for translation from English, independent back-translation, and a harmonization process, described elsewhere [7].

The CIDI-WMH involves administration of a fully structured interview schedule with broad coverage of health and mental health topics, including psychiatric and behavioral disturbances. Within this context, the CIDI-WMH includes modules that present standardized questions on the use of specific drug compounds, and questions to assess clinical features associated with drug use disorders, in conformity with the abuse and dependence definitions and criteria of the Diagnostic and Statistical Manual of Mental Disorders, Fourth Edition (DSM-IV; APA, 1994).

The CIDI-WMH module that taps the diagnostic criteria and features of "DSM-IV Drug Abuse" poses five questions (see Additional file 1). Four of these questions refer to work, social and legal problems, as well as to recurrent hazard-laden use. These four questions about the DSM-IV drug abuse construct were asked to all people who completed the CIDI-WMH drug disorders module. The fifth question asked about continued drug use despite experiencing social problems, and was conditioned on a positive answer to the social problem question.

It is of note that the CIDI-WMH poses the DSM-IV drug abuse questions in a way that make them non-specific for each of the types of drugs just mentioned (i.e., cannabis, cocaine, heroin, other IRD). This means that when multiple drugs had been used, we cannot know which specific drug accounted for the occurrence of a clinical feature. Thus, in order to focus this investigation on problems specifically related to cannabis, and to eliminate the influence of other drugs on the occurrence of these clinical features, an exclusion rule was applied. Here, for this report, the drug users under study were those whose illegal drug experiences were limited to cannabis and only cannabis (i.e., they are 'cannabis only' users).

It should also be noted that in all four surveys (i.e., NCS-R, Mexico, Colombia and NLAAS) the "DSM-IV drug abuse" clinical features module was preceded by a screening module, which was used to identify respondents who might have experienced 'clinically significant' problems with either alcohol or other drugs. This screening module was administered in Part 1 of all four surveys. It invokes a broad and non-specific assessment of 'clinically significant' problems via the following three standardized questions: a) "Did you ever use alcohol or drugs so much that your family or friends worried about you or repeatedly complained about your use?" b) "Did you ever use alcohol or drugs so much that it caused repeated arguments or problems either with your family or friends, people at work or school, or with the police?" c) "Did you ever use alcohol or drugs so much that it often interfered with your responsibilities at work, at school, or at home?." Only those participants who had tried illegal drugs and experienced at least one of these 'clinically significant' problems were asked DSM-IV drug abuse module questions.

In total, the DSM-IV drug abuse module was administered to 58 (16%) of 353 'cannabis only' (CO) users in the NLAAS, 69 (27%) of 260 CO users in Colombia, 59 (28%) of 212 CO users in Mexico, and 262 (19%) of 1,371 CO users in the NCS-R. Accordingly, the measurement theory of the research team followed a logic pertinent to the DSM-IV construct of DSM-IV disorders that first was articulated by Narrow et al. [9], and that has been discussed by Degenhardt and colleagues [10-12]. As discussed, this type of gating procedure should be highly sensitive, leaving behind very few cases of drug use disorders of 'clinical significance' as required for the DSM criteria. Accordingly, in this study, we imputed zeros when cannabis-using respondents failed to meet the 'clinical significance' criterion that is implicit in the screening module of the CIDI-WMH protocol for the assessment of the DSM-IV drug use disorder. It follows that a standard measurement assumption for these studies is that no clinically significant drug problems had occurred unless the drug user had experienced at least one of the three broad and non-specific problems assessed by the CIDI-WMH screening module described above. We will return to this assumption in the discussion section of this article. Lastly, although the NLAAS assessed cannabis-associated legal problems, the NLAAS public use dataset does not include this variable due to concern that its release might permit inadvertent identification of specific individuals, in violation of confidentiality and privacy protections.

### Procedure

Details about the coordinated sampling, project plan, and assessment approaches are available in online reports http://www.icpsr.umich.edu/cocoon/cpes/using.xml, last accessed 11 Sep 2009), and in a series of journal publications [1,7,13,14]. A brief overview of the WMHS and NLAAS research approach follows and appears in Table 1 (Panel 1).

**Table 1:** Description of the surveys conducted at each site.

Country	Survey	Sample characteristics*	Dates	Age	Sample size		Response rate, %**
					**Part1**	**Part2**	
						
Colombia	NSMH	Stratified multistage clustered area probability sample of household residents in all urban areas of the country (approximately 73% of the total national population).	2003	18-65	4,426	2,381	88
Mexico	M-NCS	Stratified multistage clustered area probability sample of household residents in all urban areas of the country (approximately 75% of the total national population).	2001-2002	18-65	5,782	2,362	77
USA	NCS-R	Stratified multistage clustered area probability sample of household residents, nationally representative.	2002-2003	≥18	9,282	5,692	71
USA	NLAAS (Latino sample)	Stratified multistage clustered area probability sample of household residents, nationally representative of Latino population with special supplements of Puerto Rican and Cuban people.	2002-2003	≥18	2,554	348	76

Abbreviations: NSMH, the Colombian National Study of Mental Health; M-NCS, the Mexico National Comorbidity Survey; NCS-R, the US National Comorbidity Survey Replication; NLAAS, the National Latino and Asian American Study of Mental Health.

*Most World Mental Health surveys are based on stratified multistage clustered area probability household samples in which samples of areas equivalent to municipalities or counties in the United States were selected in the first stage followed by 1 or more subsequent stages of geographic sampling (eg, towns within counties, blocks within towns, households within blocks) to arrive at a sample of households, in each of which a listing of household members was created and 1 or 2 people were selected from this listing to be interviewed. No substitution was allowed when the originally sampled household resident could not be interviewed. These household samples were selected from census area data in all countries. United States surveys are based on nationally representative household samples, while Colombia and Mexico are based on nationally representative household samples in urbanized areas.

**The response rate is calculated as the ratio of the number of households in which an interview was completed to the number of households originally sampled, excluding from the denominator households known not to be eligible either because of being vacant at the time of initial contact or because the residents were unable to speak the designated languages of the survey.

The WMHS and NLAAS assessment had two parts: Part 1 and Part 2. This arrangement was intended to reduce the questioning burden for the study participants, most of whom had no illegal drug experience whatsoever. Part 1 was administered to all participants and included core diagnoses of central interest. These core diagnoses encompassed basically mood and anxiety disorders (e.g., major depression, dysthymia, bipolar disorder, panic attack, generalized anxiety disorder), with some minor variations across countries. For example, the alcohol and other drug use modules, and related disorders modules, were included in Part 1 for Colombia, Mexico and the NLAAS; for the NCS-R, these modules were in Part 2.

In general, Part 2 included disorders of secondary interest to the primary sponsors of the WMHS and NLAAS (anorexia, bulimia, obsessive compulsive disorder, conduct disorder, attention deficit disorder, among others). It was administered to a probability subsample of Part 1 participants, selected via a computerized algorithm. Basically, in the NCS-R, the rules for this algorithm included meeting lifetime history criteria for any of the Part 1 core disorders, a probabilistic sample of 59% of those participants who reported behavior problems or psychiatric symptoms but failed to meet criteria for any of the Part 1 core disorders; and a probabilistic sample of 25% of all others [14,15]. These variations in probability of selection are taken into account through appropriate weights during data analysis.

### Analysis Plan

A standard three-step approach was implemented: i) explore the marginals (i.e., Tukey-style exploratory data analysis); ii) analyze/estimate; and iii) explore again (e.g., regression diagnostics; evaluate model assumptions about subgroup variation). Analyses took into account weighting for sample selection probabilities, post-stratification adjustment factors, and the multi-stage nested structure of the sampling plan. Taylor series linearization methods were used for variance estimation when appropriate.

We estimated population subgroup proportions for each clinical feature of cannabis use in each country's sample of CO users. A generalized linear model (GLM) with a logistic link function was used to study cross-national variation in the occurrence of the clinical features. Because these clinical features are expected to be inter-correlated (i.e., clustered within individuals), the generalized estimating equations (GEE) were used to bring the error structure for multiple logistic regression into conformity with regression assumptions that, if ignored, might produce erroneous scientific conclusions and reduced efficiency and precision of regression estimates [16]. However, for the assessment of variation in the log odds of continued cannabis smoking despite awareness of cannabis-caused social problems, a binary logistic regression model was used, with a subpopulation structure to highlight the subgroup of cannabis smokers who had experienced social problems.

Finally, because the analyses sometimes were based on a small number of CO users who had experienced clinically significant problems, we also applied exact methods for logistic regression [17]. Moreover, to address the notion that some of the observed associations might be strongly confounded by male sex and history of alcohol use disorders, (AUD) [6,18], we matched on sex and AUD, and then fit an exact conditional logistic regression, with covariate terms as listed above. In our samples, alcohol problems were present in 22% of CO users in Colombia, 20% in Mexico, and about 15% in both USA general population and USA Latino population. We convey the precision of the odds ratio estimates with 95% confidence intervals as well as p-values (alpha at 0.05). Analyses were performed in Stata 10.0 [19] and LogXact 8.0 [20].

## Results

Table 2 conveys the distribution by country of the 2,196 respondents who had smoked cannabis but had not engaged in illegal use of cocaine, heroin, or other internationally regulated drugs (IRD); as noted above, virtually all had consumed alcohol and 2/3rds had smoked tobacco. The cumulative occurrence of CO use was larger in the USA population at large (NCS-R), followed by the Latino population living in the USA (NLAAS), Colombia, and then Mexico, with statistically robust differences in that order (p < 0.05). Given that these differences might vary as a function of the respective cumulative incidence for cannabis smoking, regardless of the illegal use of other IRD, we estimated the relative proportion of CO users among all cannabis users. Thus, in the USA general population (NCS-R), about 51% 'cannabis ever' users had used cannabis exclusively (95% confidence interval, CI = 49, 54), which was similar to the corresponding 50% estimate for the USA Latino population (NLAAS; 95% CI = 45, 56). In contrast, among 'cannabis ever' users from Colombia, 63% used cannabis exclusively (95% CI = 57, 69), and for Mexico the corresponding estimate was 57% (95% CI = 51, 64).

**Table 2:** Estimated cumulative occurrence (%) of cannabis smoking with no other illegal drug use.

	Colombia			Mexico			Latinos USA (NLAAS)			USA (NCS-R)		
	
	N*	n^¥^	%** (95%CI)	N*	n^¥^	%** (95%CI)	N*	n^¥^	%** (95%CI)	N*	n^¥^	%** (95%CI)
All persons	4,426	260	7 (6, 8)	5,782	212	4 (4,5)	2,554	353	15 (13, 17)	5,692	1,371	22 (20, 23)
Sex												
Male	1,700	195	11 (9, 13)	2,285	184	9 (7, 10)	1,127	200	20 (17, 22)	2,382	580	23 (20, 25)
Female	2,726	65	3 (2, 4)	3,497	28	1 (<1, 1)	1,427	153	10 (8, 13)	3,310	791	21 (19, 23)
Age Group												
18-29	1,431	99	7 (6, 9)	2,060	76	5 (3, 6)	731	161	22 (17, 27)	1,371	408	28 (25, 31)
30-44	1,735	94	6 (4, 7)	2,236	92	5 (4, 7)	931	125	12 (10, 15)	1,826	508	27 (24, 29)
45-54	730	49	8 (5, 11)	840	26	3 (1, 5)	394	46	16 (12, 19)	1,123	319	26 (22, 30)
>55	530	18	6 (2, 10)	646	18	3 (1, 5)	498	21	6 (3, 9)	1,372	136	9 (7, 11)

* Unweighted number of participants (i.e., simple count).

^¥ ^Unweighted number of cannabis-only users (i.e., simple count).

** Indicates weighted data with Taylor series linearization for variance estimation.

Data from WMH and CPES Surveys in Colombia, Mexico, and USA, 2002-3.

In testing the hypothesis that the proportion of CO users among the 'cannabis ever' users was similar across the four country populations, we found robust differences only in the contrast of Colombia versus the USA. Specifically, after adjusting for age and sex, the odds of being a CO user among the Colombian 'cannabis ever' users was 1.6 times the NCS-R-estimated odds of being a CO user in the USA (i.e., adjusted odds ratio, aOR = 1.6; 95% CI = 1.2, 2.1; p = 0.003). Similarly, after adjusting for age and sex, the odds of being a CO user among the Colombian 'cannabis ever' users was 1.7 times the NLAAS-estimated odds of being a CO user among USA Latino 'cannabis ever' users (i.e., aOR = 1.7; 95% CI = 1.2, 2.4; p = 0.003). There were no robust differences in other pairwise comparisons (i.e., Mexico versus USA general population, Mexico versus USA Latinos, and USA Latinos versus USA general population).

Table 2 also shows the estimated cumulative occurrence of CO use specific for sex and age across the populations studied. There were consistently more CO users among men than among women in Colombia, Mexico and the USA Latino population. However, in the USA general population (NCS-R), there was no male-female variation in estimated cumulative occurrence of 'cannabis only' use. In addition, for Colombia and Mexico, there was little age-related variation in occurrence of CO, whereas for the USA Latinos and general population the estimated cumulative occurrence of CO use was less frequent among people age 55 years and older.

The population-specific estimated proportions presented in Table 3 convey that the experience of cannabis-related clinical features was quite infrequent among CO users in all three countries, with weighted frequencies ranging from 1% to 5%. In general, recurrent hazard-laden cannabis smoking (e.g., driving while intoxicated) and social problems attributed to cannabis seem to be the most frequent clinically significant cannabis-related problems, followed by cannabis-related work problems. Cannabis-related legal problems were quite infrequent (Table 3).

**Table 3:** Estimated cumulative occurrence of each clinical feature related to abuse among 'cannabis only' users, by country population.

Clinical Features	Colombia		Mexico		Latinos USA (NLAAS)		USA (NCS-R)	
	
	n^¥^	% (95%CI)*	n^¥^	% (95%CI)*	n^¥^	% (95%CI)*	n^¥^	% (95%CI)*
Recurrent hazard-laden smoking	9	3 (1, 5)	7	3 (<1, 5)	15	6 (3, 9)	59	3 (2, 4)
Social problems	13	5 (2, 7)	8	3 (<1, 5)	5	2 (<1, 3)	27	1 (1, 2)
Continued smoking despite experiencing these problems^‡^	9	4 (1, 6)	6	2 (<1, 4)	4	2 (<1, 3)	22	1 (1, 2)
Work problems	8	2 (<1, 4)	6	2 (<1, 4)	5	1 (<1, 3)	24	1 (<1, 2)
Legal problems	6	2 (<1, 4)	3	1 (<1, 2)	Not available**		5	Not estimated

^¥ ^These are simple counts of cannabis-only smokers who experienced each clinical feature as listed by row, and by country, before weighting the data. The small counts constrained what can be done in relation to original plans for more complex modeling, and placed limits on statistical power and precision.

* The estimated percents and 95% CI are based on application of the survey weights and estimation as described in the methods section.

** Data on 'Legal problems' are not publicly available for the NLAAS participants (not released to reduce likelihood of a confidentiality violation).

^‡^This question was asked only to those who gave a positive answer to the query regarding the experience of social problems.

Data from WMH and CPES Surveys in Colombia, Mexico, and USA, 2002-3.

In the comparison of Colombia CO users with USA CO users, living in Colombia was associated with greater occurrence of cannabis-related social and legal problems, even with statistical adjustment for sex and age. In specific, CO users in Colombia were more likely to have experienced 'social problems' (aOR = 3.0; 95% CI = 1.4, 6.3; p = 0.004) and 'legal problems' (aOR = 9.7; 95% CI = 2.7, 35.2; p = 0.001), as compared to CO users in the USA general population. There were no such associations for work problems and recurrent hazard-laden use. In addition, there was some evidence that the USA Latino CO users tended to be engaged in recurrent hazard-laden use more often than those in Mexico. However, this finding failed to be statistically significant by the conventional standard of p < 0.05 after adjusting by sex and age (aOR = 2.9; 95% CI = 1.0, 8.5; p = 0.051). There was no USA Latino v. Mexico variation in the other cannabis problems under study.

Large confidence intervals for the 'legal problems' estimates in the Colombia-USA contrast implies instability of estimates due to the small numbers of CO users who experienced this type of problem. Due to the small numbers, we turned to exact logistic regression and re-estimated the odds ratio, adjusting for sex and age. Evidence from this 'exact' analysis helps confirm that, in Colombia, CO users were more likely to experience 'legal problems' than CO users in the USA general population (OR = 4.4; 95% CI = 1.1, 19.6; exact p = 0.039). To provide additional control over possible confounding by a past history of alcohol use disorders (AUD) and sex, we formed risk sets after matching on AUD and sex such that everyone within each separate risk set had the same values of the AUD and sex variables. Using the 'exact' conditional logistic regression model with these AUD- and sex-matched risk sets, the resulting age-adjusted odds ratio estimate supports an inference of excess occurrence of cannabis-related legal problems in Colombia versus in the USA general population (OR = 4.9; 95% CI = 1.1, 23.3; exact p = 0.034).

Finally, for assessment of variations across the study populations in the occurrence of continued use of cannabis despite the user's awareness of social problems due to cannabis, the binary logistic regression model was used, with a restriction to CO users who had experienced cannabis-related social problems. This analysis disclosed no statistically robust variation across populations in relation to that clinical feature (p-values > 0.05 for all pairwise comparisons).

## Discussion

This study's main findings can be summarized as follows. Among adults in the USA, about half of the cannabis smokers have used other illegal or internationally regulated drugs, whereas this was somewhat less common in Colombia and Mexico, where 'cannabis only' use was more common. We also found that the cannabis problems were experienced rather infrequently by the CO users, which followed a similar pattern for all three countries. However, there was an exception to that general rule: CO users in Colombia were more likely to experience cannabis-related social and legal problems as compared to CO users in the USA general population. Regrettably, one limitation of this study involves data on cannabis-related 'legal problems,' which were assessed in the NLAAS, but the NLAAS principal investigators did not release these values due to concern about inadvertent violation of confidentiality and incomplete privacy protection (e.g., if the survey data were matched to publicly available data on cannabis offenses). For this reason, we were unable to compare Colombia's estimates with those of the US Hispanic subgroups surveyed for the NLAAS, which would have been especially interesting, given observed greater occurrence of cannabis-associated legal problems in Colombia.

Another finding of potential interest involves recurrent hazard-laden cannabis smoking, and the observation that USA Latino CO users tended to engaged in recurrent hazard-laden use more often than those in Mexico. To the best of our knowledge, there is neither strong theory nor prior evidence on recurrent hazard-laden use from prior cross-national studies of this type, which might have been used to establish a set of Bayesian priors for this contrast, or to calibrate statistical power for a more complete balance of Type I and Type II error. In this context, readers may wish to note that p = 0.051 is not too distant from the conventional standard of 0.05. A slightly larger sample might well have produced p < 0.05 with an effect estimate of this size; this is a contrast that may deserve future investigation and confirmation in future epidemiological studies.

Before detailed discussion, several other limitations deserve to be mentioned. Despite the size of the overall samples in each place, there were relatively small numbers of CO users who had experienced cannabis problems, according to the CIDI-WMH assessment. In consequence, no more than a handful of covariate terms could be introduced in the statistical models; statistical power and precision also were limited. Moreover, there were too few CO users in the samples to permit a focused study on the recent-onset cannabis smokers as has been done elsewhere [8]. This constraint also thwarted any detailed probing of male-female differences, or research on issues of migration and acculturation, as one might wish to examine in relation to the experience of specific subgroups of Spanish-speakers in these samples (e.g., Mexico, Colombia, or other country of origin for the USA Latinos). In addition, the self-report character of the assessment of cannabis-related problems can be expected to introduce some measurement error, which we suspect might be manifest as 'under-reporting' of the clinical features if not cannabis smoking per se, as discussed elsewhere [4,11].

One other possible limitation has to do with design issues as described in our methods section, especially the CIDI-WMH focus on 'clinically significant' cannabis problems. Readers interested in more details about this issue are referred to original work by Narrow and colleagues [9] and to more recent contributions by Degenhardt and colleagues [10-12], in which it has been found that this particular CIDI-WMH approach seems to have had no appreciable influence when the task is to estimate the occurrence of DSM-IV cannabis dependence and related problems.

A last limitation is the narrow range of cannabis experiences covered in the standardized diagnostic assessments. In future global health research on cannabis-associated experiences, it should be possible to extend the coverage of cannabis experiences in two directions: (a) toward positive and possible health-enhancing experiences associated with cannabis consumption (e.g., of the type discussed by Griffiths et al.(2006) [21], as well as appetite-promoting or nausea-reducing effects discussed in the context of medicinal use of cannabis products); and (b) a more complete coverage of craving and the obsession-like or compulsion-like experiences associated with cannabis dependence syndromes. The global prevalence of cannabis smoking provides ample justification for this type of focused inquiry.

Despite caveats such as these, this study has the strength of generally comparable data gathering with a standard design and implementation protocol across countries as part of the WMHS initiative. Each population sample in the three countries was obtained through similar multi-stage sampling methods with good to excellent participation levels. Each participant completed standardized survey assessments of high quality.

Against this background of limitations and strengths, this study shows that CO users represent in general a substantial majority of all cannabis users in the studied countries, which justify a focused look at these CO users with respect to clinical features and problems associated with the drug disorders. Separately, and with evidence from more countries, WMHS collaborators are following up this investigation with a look at all cannabis users, and are estimating the influence of polydrug use on the occurrence of these clinical features, but these analyses introduce a good bit of complexity in the cross-national research context, as there is a good deal of heterogeneity in the profile of internationally regulated drugs used, plus large between-country variations in the police responses to the different drug compounds.

Also, this study found that as a general rule the estimated experience of CO users with respect to 'clinically significant' cannabis problems did not vary appreciably across the samples, even though there is a wide variation in the occurrence of cannabis use across these national boundaries within the Americas. The exception to the rule was found in the Colombia-USA contrast, with an excess occurrence of cannabis-related legal and social problems in Colombia. We speculate that the observed variation might be traced to differences in the context in which cannabis is consumed in each country, such as whether alcohol is being consumed concurrently. Concurrent use of cannabis with alcohol has been described in the epidemiological literature as the most common pattern of polydrug use, and has been related to excess occurrence of social and psychological consequences [6,22], and also to behavioral patterns that might promote adverse consequences, such as attending parties more often [6,18]. Our use of AUD-matching and the exact conditional logistic regression was motivated by an effort to probe into this possibility, but AUD does not encompass the concept of concurrent or simultaneous alcohol-cannabis use, so this issue must be left for future investigations, with deliberate assessment of concurrency and simultaneity of alcohol and other drug use, as recommended elsewhere [23].

When introducing our study in this paper's 'Background' section, we also mentioned the possibility that the THC content of smoked cannabis might vary considerably across countries. We also mentioned other cross-country and within-country variations in cannabis ingestion practices. For example, our colleagues from western Europe, Turkey, and Egypt were surprised to learn that in the Americas and in New Zealand, the cannabis typically is smoked by itself, and not in cannabis-tobacco formulations (with the exception of American 'blunts' - namely, hollowed out tobacco cigars into which the cannabis is inserted and smoked). Our colleagues from India were surprised at the narrow range of cannabis preparations generally available in the Americas, and brought to our attention many different teas, cakes, and other formulations for cannabis in their country. The World Mental Health Surveys were not focused specifically upon cannabis, and gathered essentially no information about these details of cannabis consumption. In future cross-national research focused specifically upon cannabis consumption, it may be possible to study cannabis-related experiences with more careful probing into possibilities that different cannabis experiences depend upon these cannabis ingestion practices.

The evidence of this study also draws attention to some unanswered questions about the experience of Latinos living in the USA. In the WMH research, with similar survey methods used for all places, it was found that 30% of US Latinos had smoked cannabis (unpublished National Latino and Asian American Study, NLAAS, estimate derived for this study report), which is a value about halfway between what was found for Colombia and Mexico (10-11% and 7-8%, respectively) and the USA (42-43%) [14]; nonetheless, there was no appreciable variation in the experience of clinically significant cannabis problems in the USA general population versus US Latino contrast. In future research with larger sample sizes and a focus on these research issues, it should be possible to examine these interesting cannabis patterns in relation to (a) birth cohort variations, (b) time elapsed since migration into the USA (including the USA-born Latinos), other issues of acculturation, assimilation, and adaptation, as has been done in relation to the epidemiology of cardiovascular diseases [24,25] and cancer [26,27]. Indeed, Borges and colleagues studied use of alcohol and other psychoactive drugs among residents of Mexico who had been in the USA at least once and then had returned to Mexico, discovering that this subgroup had greater drug involvement when compared to the subgroup of Mexicans without USA experience stratified or holding constant whether they had close family members who had migrated to the USA [28].

## Conclusion

Despite methodological limitations, this investigation has shed new light on cross-national variation in the occurrence of cannabis smoking and related problems among community-dwelling adult residents of three countries of the Americas and among Latino residents in the USA. In these places, a majority of cannabis smoking adults had not engaged in illegal use of cocaine, heroin or other drugs, and among these 'cannabis only' users a very small minority (<10%) had developed clinically significant clinical features that we associate with cannabis use disorders as diagnosed by contemporary case definitions. As a general rule, there was no appreciable cross-national variation in the occurrence of these clinical features among 'cannabis only' users, which suggests that the wide cross-national variability of the cumulative incidence of cannabis use disorders seen in different world reports may be due more to quantitative differences in the number of people involved with this drug across countries, rather than qualitative differences in the experience of each of the cannabis problems under study. Our finding that Colombia 'cannabis only' users had more often experienced cannabis-associated social and legal problems, as compared to 'cannabis only' users in the USA general population deserves further investigation. Our research group has offered speculations with respect to possible explanations for the observed Colombia-USA difference, but future studies will be required to confirm and to produce more definitive evidence about the observed difference.

## Abbreviations

aOR: Adjusted odds ratio; AUD: Alcohol Use Disorder; CF: Clinical feature; CI: Confidence Interval; CIDI: Composite International Diagnostic Interview; CO:Cannabis-only; CPES: Collaborative Psychiatric Epidemiology Surveys; DSM-IV: Diagnostic and Statistical Manual of Mental Disorders, Fourth Edition; GEE: Generalized estimating equation; GLM: Generalized linear regression; IRD: Internationally regulated drugs; LSD: Lysergic acid diethylamide; NCS-R: National Comorbidity Survey; NLAAS: National Latino and Asian American Study; OR: Odds ratio; SP: Survey population; THC: delta-9 tetrahydrocannabinol; UN: United Nations; USA: United States; USNCSR: United States National Comorbidity Survey Replication; WHO: World Health Organization; WMHS: World Mental Health Surveys.

## Competing interests

The authors declare that they have no competing interests.

## Authors' contributions

MMM, JPV adapted the WMHS Consortium protocol for their countries and collected those data. JCA worked with Harvard University colloborators early in the design of the WMHS Consortium protocol, and continues as leader of the WMHS drug dependence work group; a Harvard - University of Michigan team gathered the US data. FF and JCA designed the analysis; FF completed the analyses with consultation from JCA, MR and SM. FF drafted the paper and prepared its tables, with all authors as participants in revising and polishing the drafts, interpreting the data, and drawing conclusions. All authors read and approved the approved the final manuscript.

## Pre-publication history

The pre-publication history for this paper can be accessed here:

http://www.biomedcentral.com/1471-2458/10/152/prepub

## Supplementary Material

Additional file 1 **Appendix**. Composite International Diagnostic Interview for the World Mental Health Surveys (CIDI-WMH) Click here for file
