# Supplementary material for: Cross-national differences in clinically significant cannabis problems: epidemiologic evidence from 'cannabis-only' smokers in the United States, Mexico, and Colombia
Source: BMC Public Health. 2010 Mar 23;10:152. doi: 10.1186/1471-2458-10-152 (PMC2863162; doi:10.1186/1471-2458-10-152)
Supplement: Additional file 1 — Appendix. Composite International Diagnostic Interview for the World Mental Health Surveys (CIDI-WMH) [file 1471-2458-10-152-S1.DOC]

Appendix

Composite International Diagnostic Interview for the World Mental Health Surveys (CIDI-WMH)

| **Clinical Feature** | **CIDI-WMH question** |
| --- | --- |
| Recurrent hazard-laden smoking | “*Were there times in your life when you were often under the influence of [marijuana or hashish] in situations where you could get hurt, for example when riding a bicycle, driving, operating a machine, or anything else?”* |
| social problems | “*Was there a time in your life when your use of [marijuana or hashish] caused arguments or other serious problems with your family, friends, neighbors, or co-workers?”* |
| Continuing to smoke despite social problems* | “*Did you continue to use it even though it caused problems with these people?”* |
| Work problems | “*Was there a time in your life when the use of [marijuana or hashish] frequently interfered with your work or responsibilities at school, on a job or at home?”* |
| Recurrent legal problems** | “*Were you more than once arrested or stopped by the police because of driving under the influence of [marijuana or hashish] or because of your behavior while you were high?”* |
| *This question was asked only to those who gave a positive answer to the query regarding the experience of social problems. | |
| ** The USA-Latino public use dataset suppressed the legal problems data in order to protect identities of respondents. Otherwise, all cross-survey methods and data were comparable. | |
